# Supplementary figures and images for: Characterization of neurogenic niches in the telencephalon of juvenile and adult sharks
Source: Brain Struct Funct. 2020 Feb 15;225(2):817–39. doi: 10.1007/s00429-020-02038-1 (PMC7046584; doi:10.1007/s00429-020-02038-1)

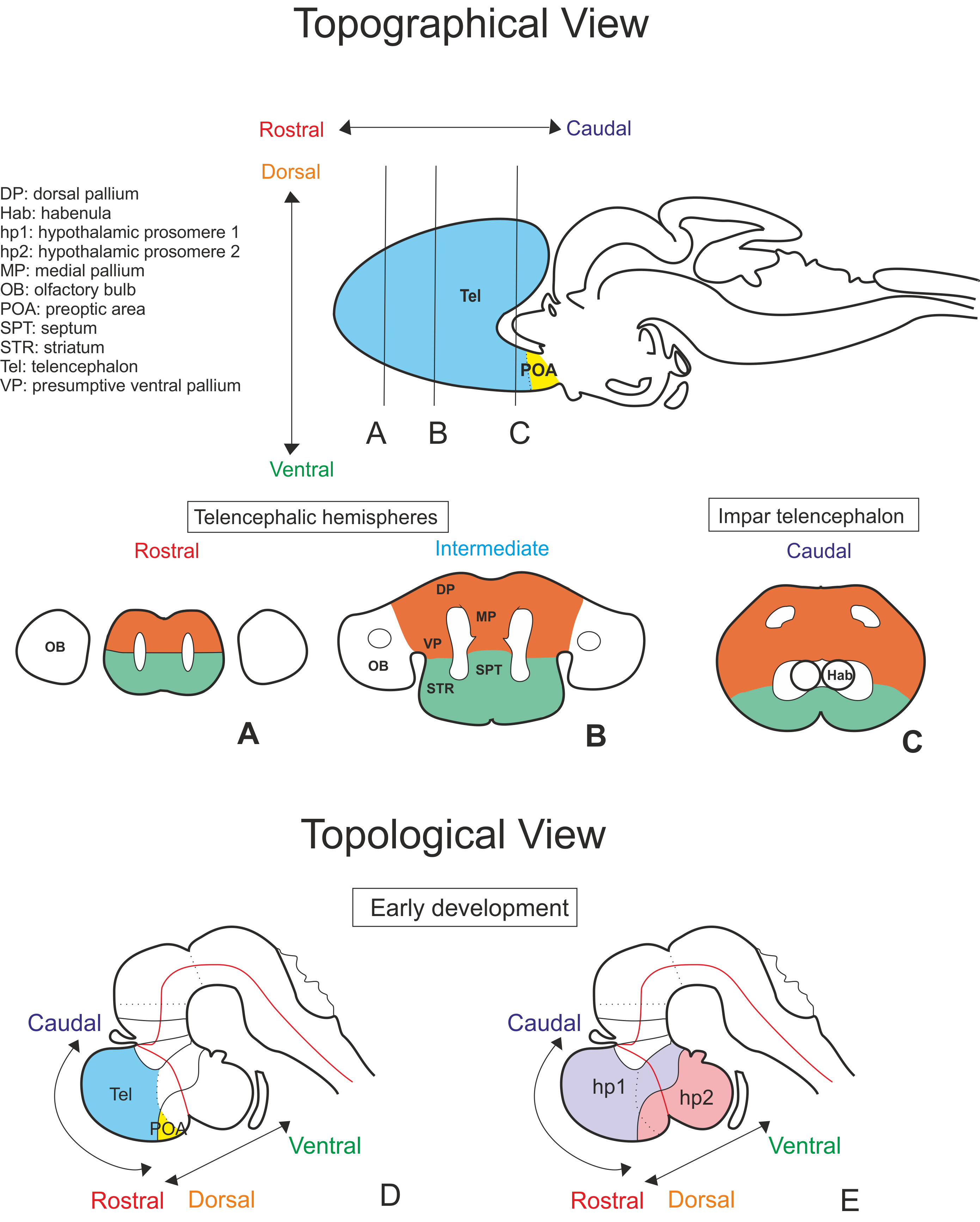

Supplement: Supplementary file 1 — Supplementary Figure 1. Schema showing the anatomy of the telencephalon of the catshark, showing topographic (A-C) and topologic (D-E) views. In the schemas from telencephalic transverse sections, pallium is represented in red and subpallium is represented in green. Schemas based on Smeets et al. 1983 and Santos-Durán et al. 2015 (TIF 37683 kb) [file 429_2020_2038_MOESM1_ESM.tif]

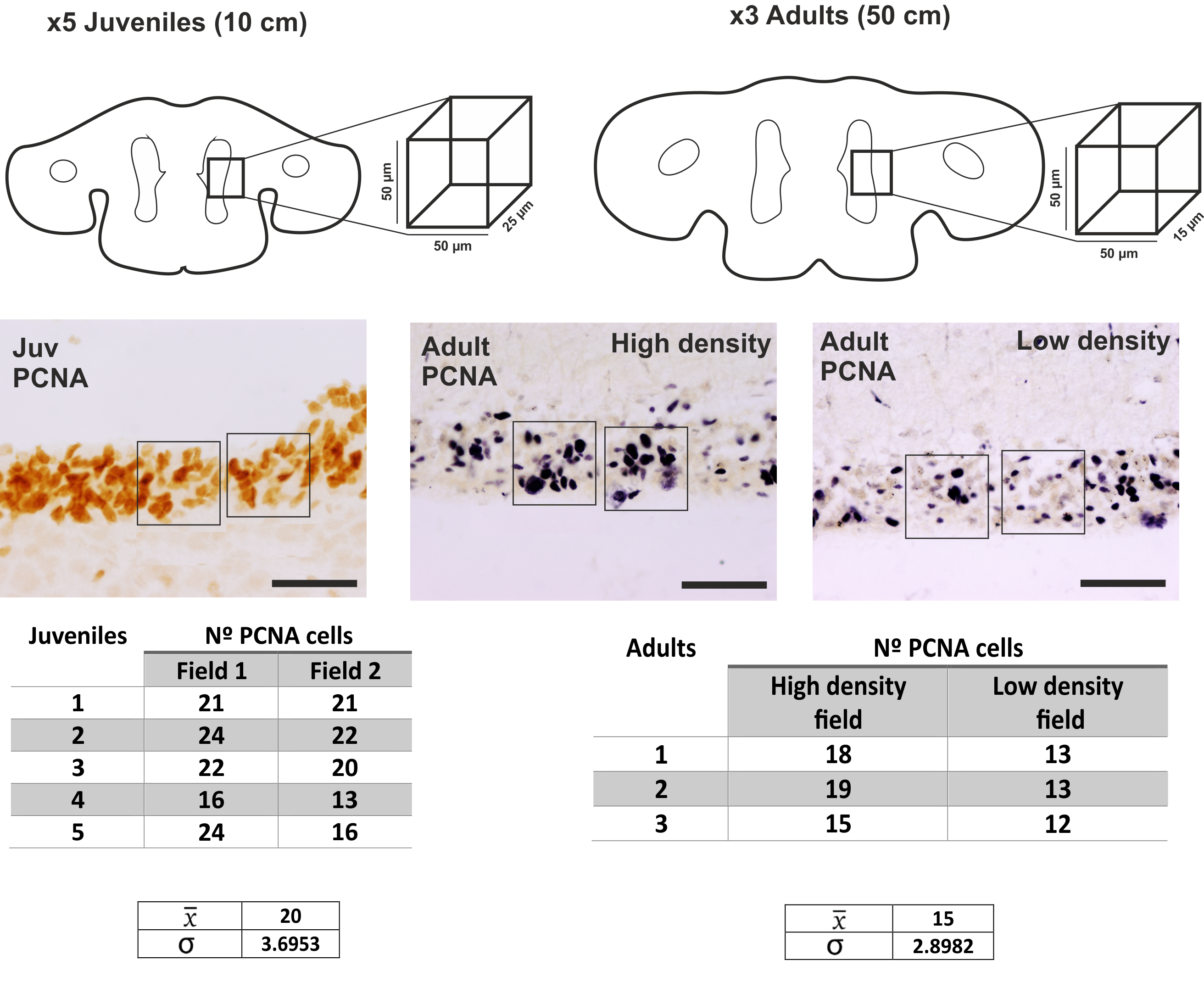

Supplement: Supplementary file 2 — Supplementary Figure 2. Schema from transverse sections of the telencephalon of juveniles (Juv) and adults representing the box area used for cell counting of PCNA-ir cells in the ventral pallium, the number of samples used and the average and standard deviations. Numbers of cells were quantified in the ventral pallium, where high numbers of PCNA-ir cells are observed in juveniles with respect to other telencephalic regions (TIF 22338 kb) [file 429_2020_2038_MOESM2_ESM.tif]

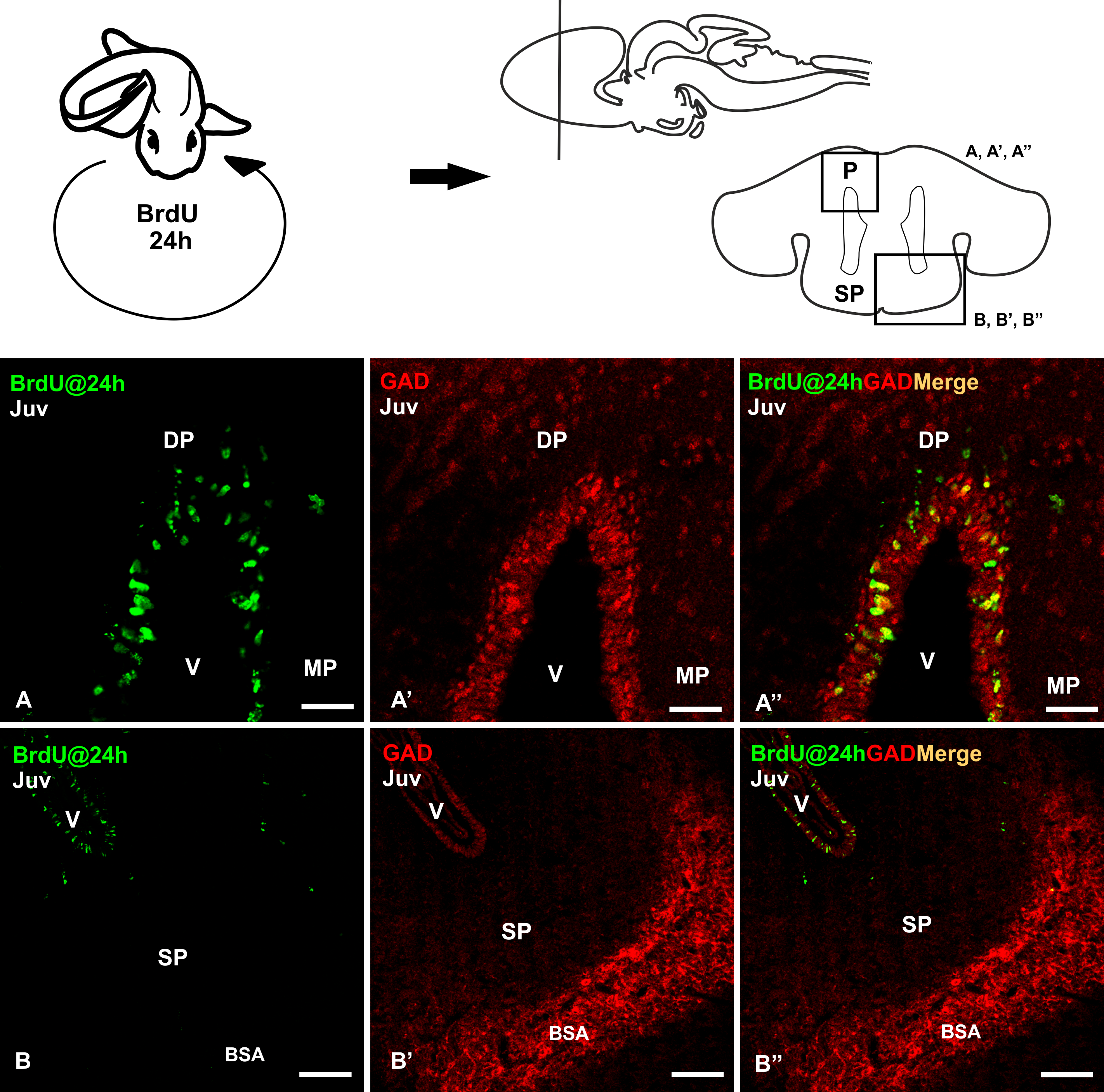

Supplement: Supplementary file 3 — Supplementary Figure 3. Schemes and photomicrographs after BrdU 24h pulses in the telencephalon of catshark juveniles (Juv) showing double labelled cells for BrdU and GAD in the VZ of the dorsal and medial pallium (A-A’’) and labelled cells for BrdU but not for GAD in the subpallial ventricular zone (B-B´´). Note the high expression of GAD in the subpallium at the level of the basal superficial area (B-B’’). Scale bars: 50 µm (A- A’’); 200 µm (B-B´´). Abbreviations: BSA, basal superficial area; DP, dorsal pallium; MP, medial pallium; SP, subpallium; V, ventricle (TIF 31265 kb) [file 429_2020_2038_MOESM3_ESM.tif]
